# Role of mTOR Inhibitors in Kidney Disease

**DOI:** 10.3390/ijms17060975

**Published:** 2016-06-21

**Authors:** Moto Kajiwara, Satohiro Masuda

**Affiliations:** Department of Pharmacy, Kyushu University Hospital, 3-1-1 Maidashi, Higashi-ku, Fukuoka 812-8582, Japan; motokaji@jsd.med.kyushu-u.ac.jp

**Keywords:** mTOR inhibitor, kidney, renal cancer, diabetic nephropathy, kidney transplantation

## Abstract

The first compound that inhibited the mammalian target of rapamycin (mTOR), sirolimus (rapamycin) was discovered in the 1970s as a soil bacterium metabolite collected on Easter Island (Rapa Nui). Because sirolimus showed antiproliferative activity, researchers investigated its molecular target and identified the TOR1 and TOR2. The mTOR consists of mTOR complex 1 (mTORC1) and mTORC2. Rapalogues including sirolimus, everolimus, and temsirolimus exert their effect mainly on mTORC1, whereas their inhibitory effect on mTORC2 is mild. To obtain compounds with more potent antiproliferative effects, ATP-competitive inhibitors of mTOR targeting both mTORC1 and mTORC2 have been developed and tested in clinical trials as anticancer drugs. Currently, mTOR inhibitors are used as anticancer drugs against several solid tumors, and immunosuppressive agents for transplantation of various organs. This review discusses the role of mTOR inhibitors in renal disease with a particular focus on renal cancer, diabetic nephropathy, and kidney transplantation.

## 1. Introduction

**Figure 1:**
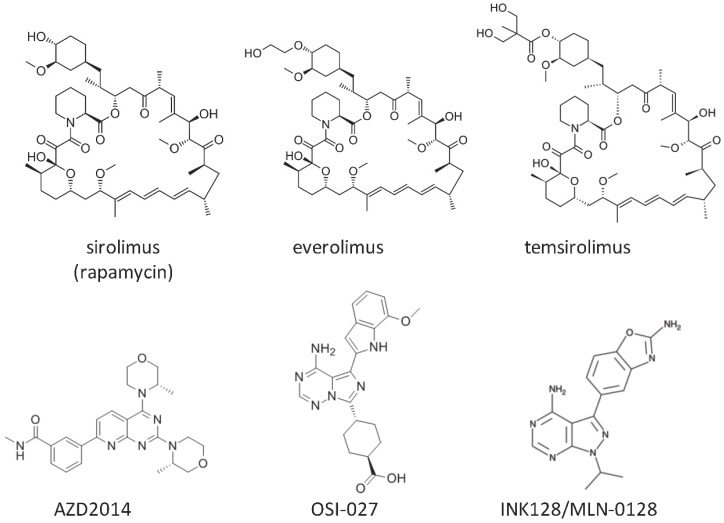
Chemical structures of mammalian target of rapamycin (mTOR) inhibitors.

**Figure 2:**
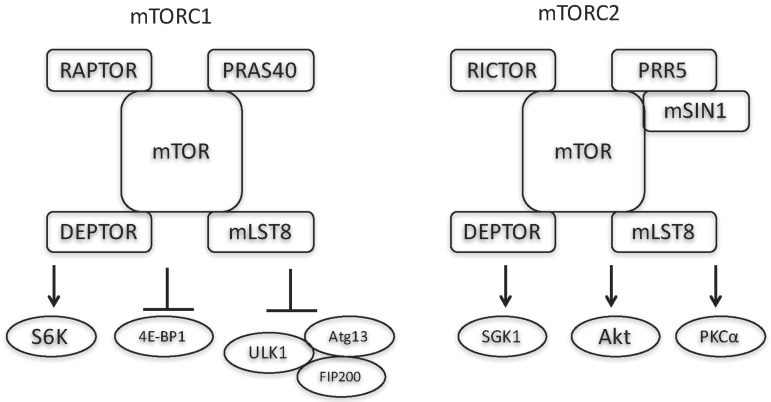
Structures of mTORC1 and mTORC2. mTORC1 phosphorylates substrates including S6K, 4E-BP1, and the unc-51-like kinase 1/mammalian autophagy-related gene 13/focal adhesion kinase family-interacting protein of 200 kDa (ULK1/Atg13/FIP200) complex. mTORC2 phosphorylates substrates including serum/glucocorticoid regulated kinase (SGK) 1, Akt, and protein kinase C-α (PKCα).

The first compound inhibiting mammalian target of rapamycin (mTOR), sirolimus (rapamycin), was identified in the 1970s from a soil bacterium collected on Easter Island (Rapa Nui) (Figure 1) [1]. Sirolimus showed antifungal, antitumor, and immunosuppressive effects [1]. Beginning by the finding of two targets of rapamycin (TOR)1 and TOR2 in the yeast cells in 1991, detailed mechanisms underlying the antiproliferative activity of sirolimus had been elucidated [1,2]. In 1994, the mammalian counterparts of the yeast TOR complexes were revealed as mTOR complex 1 (mTORC1) and mTORC2 [2,3]. mTORC1 comprises mTOR, mammalian lethal with SEC13 protein 8 (mLST8), proline-rich Akt substrate of 40kDa (PRAS40), and regulatory-associated protein of mTOR (RAPTOR) (Figure 2) [1]. mTORC2 consists of mTOR, mLST8, proline-rich protein 5 (PRR5), mitogen-activated protein kinase-associated protein 1 (mSIN1), and sirolimus-insensitive companion of mTOR (RICTOR) (Figure 2) [1].

**Figure 3:**
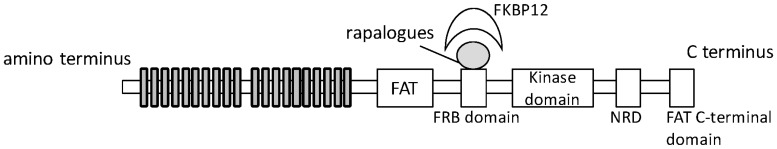
Interaction of mTOR, rapalogues, and 12-kDa FK506- and rapamycin-binding protein (FKBP12) **m**TOR contains 2549 amino acids. Rapalogues, mTOR inhibitors, form a complex with FKBP12 that binds to the FKBP-rapamycin binding (FRB) domain [4].

Sirolimus and its analogues (rapalogues) exert their effects by inhibiting mTORC1 and downstream phosphorylation of its substrates. mTOR includes several conserved motif domains (Figure 3). Rapalogues form complexes with FK506-binding protein of 12 kDa (FKBP12/FKBP1A), which binds to the (FKBP-rapamycin binding) FRB domain (Figure 3) [4]. Representative mTORC1 substrates are S6 kinase, eukaryotic translation initiation factor 4E-binding protein 1 (4E-BP1), and unc-51-like kinase 1/mammalian autophagy-related gene 13/focal adhesion kinase family-interacting protein of 200 kDa (ULK1/Atg13/FIP200). S6 kinase is activated by mTORC1 to promote mRNA translation through phosphorylation of ribosomal protein S6 [5]. 4E-EB1 inhibits mRNA translation by interrupting formation of the 5’-cap-binding protein eukaryotic initiation factor 4E (eIF4E) complex, which is needed for the initiation of cap-dependent translation, and mTORC1-mediated phosphorylation of 4E-EB1 detaches it from eIF4E, thereby promoting cap-dependent mRNA translation [5]. mTORC1-mediated phosphorylation inhibits the activity of ULK1/Atg13/FIP200, a kinase complex necessary for autophagy [5]. In addition to these roles in protein synthesis and autophagy, mTORC1 is also involved in lipid synthesis, energy metabolism, and tumorigenesis [5]. In contrast to mTORC1, mTORC2 is considered insensitive to sirolimus because FKBP12-sirolimus does not bind to mTORC2 [6,7]. However, a recent study revealed that long-term sirolimus treatment reduces mTORC2 signaling in certain cell types [8,9]. mTORC2 is involved in cell survival/metabolism and cytoskeletal organization, and its representative substrates include Akt, serum and glucocorticoid-induced protein kinase 1 (SGK1), and protein kinase C-α (PKCα) [5].

Sirolimus and its derivatives, everolimus and temsirolimus, are common mTOR inhibitors used in clinical settings (Figure 1) [10,11]. Both sirolimus and everolimus are formulated as tablets, but the solubility and bioavailability of everolimus are superior to those of sirolimus due to replacement of a hydrogen atom at position 40 by a 2-hydroxyethyl chain [12]. The anticancer and immunosuppressive effects of sirolimus and everolimus have been well investigated. Temsirolimus, a soluble ester of sirolimus formed by esterification of a hydroxyl group at position 42, was developed as an intravenous drug for the treatment of renal cell carcinoma [11]. However, these rapalogues have weak inhibitory effects on mTORC2, which is an Akt S473 kinase involved in cancer development. Therefore, ATP-competitive inhibitors of mTOR targeting both mTORC1 and mTORC2 have been developed and examined in clinical trials as a new class of anticancer drugs [13]. Although AZD2014, OSI-027, and INK128/MLN-0128 were examined in clinical trials, only INK128/MLN-0128 moved on to phase II clinical trials including renal cancer (Figure 1).

## 2. Effect of mTOR Inhibitors on Infection after Kidney Transplantation

John Cunningham (JC) virus, BK virus, and simian vacuolating (SV) 40 are polyomavirus pathogens in humans [14]. Among them, the BK virus is considered a main cause of nephropathy [14]. Most adults (>90%) are BK virus carriers who acquired BK virus during childhood [15]. BK virus-associated nephropathy is an opportunistic infection that affects up to 10% of kidney transplant recipients [15]. BK virus-associated nephropathy usually occurs within one year after transplantation, and up to 50% of kidney transplant patients with BK virus-associated nephropathy suffered graft loss [15]. Renal transplant recipients with BK virus nephropathy showed higher viral load in plasma than those without nephropathy [16]. Post-transplant immunosuppressive treatment is acknowledged as a potential risk factor for BK virus-associated nephropathy.

An early study suggested that BK virus infection and progression of the impairment of kidney grafts depended on the net state of immunosuppression and not the type of immunosuppressive drug [16]. However, recent studies showed the suppressive efficacy of mTOR inhibitor on the onset of BK virus-associated nephropathy. Everolimus and low-dose cyclosporine treatment resulted in lower incidence of BK virus-associated viremia and nephropathy compared with mycophenolic acid and standard-dose cyclosporine treatment in kidney transplant patients [17]. It was reported that the incidence of patients who underwent BK virus therapy among discharged patients receiving the cyclosporine-based maintenance dosage regimen was small in comparison with those receiving the tacrolimus-based maintenance dosage regimen, leading to speculation that cyclosporine inhibited the replication of BK virus via cyclophilin A and nuclear factor of activated T cells (NFATC) [18,19]. Similar to cyclosporine, *in vitro* studies showed that sirolimus impaired BK virus replication, inhibiting mTOR-SP6-kinase activity through an FKBP-12 pathway, but tacrolimus activated BK virus replication [20,21]. Furthermore, large-registry retrospective cohort analysis showed that the incidence of BK virus replication during the 24-month course after kidney transplantation was significantly lower in discharged patients using of mTOR inhibitors compared to those without mTOR inhibitors [19].

To confirm the clinical effect of mTOR inhibitors on BK virus infection, a prospective trial was carried out. A pilot single-center, randomized, open-labeled trial comparing the safety and efficacy of 50% reduction of mycophenolate mofetil (MMF) with the addition of everolimus (*n* = 20) *vs.* a 50% reduction of MMF (*n* = 20) in kidney transplant recipients with new onset of BK viruria >1 × 10^6^ copies/mL and/or viremia >500 copies/mL was reported at the 2015 American Transplant Congress during 2–6 May, 2015 in Philadelphia [22]. The primary endpoint was a >50% reduction of BK viruria and/or clearance of viremia at three months post-randomization. At three months post-randomization, no significant difference of reduction in BK viremia was seen between the 50% reduction of MMF with the addition of everolimus group (66.6%) and 50% reduction of MMF group (48.7%) (*p* = 0.3) [22]. A further clinical trial including more patients, and publication as an original article is needed to verify the suppressive effect of everolimus against BK virus replication. 

Although the global population is commonly (40%–70%) infected with cytomegalovirus (CMV) during childhood [23], immunocompetent individuals with CMV have no symptoms. Among kidney transplant patients, however, CMV is associated with risk of acute rejection, allograft dysfunction, end-organ disease, and mortality [23]. Immunosuppressive therapy is known as a risk factor for CMV infection and disease in kidney transplant recipients [23]. In contrast to BK virus nephropathy, it was reported that the cyclosporine regimen is related to increased CMV infection and disease incidence in kidney transplant recipients [24,25]. Among immunosuppressants, mTOR inhibitors sirolimus and everolimus might decrease the incidence and severity of CMV infection in kidney transplant recipients [23]. To proliferate, CMV requires activated mTOR in host cells. Inhibition of mTORC1 prevented the accumulation of CMV protein, and the inhibition effects were more potent immediately after CMV infection than at later time points [23].

## 3. Urinary Microtubule-Associated Protein 1 Light Chain (LC) 3: A Potential Biomarker for mTOR Inhibition in the Kidneys

Tubular atrophy and interstitial fibrosis are the final common steps in the progression of chronic kidney disease [26]. It was reported that sirolimus reduced interstitial fibrosis and glomerular sclerosis after kidney transplantation in patients with chronic allograft nephropathy [27,28]. In addition, a causal link between the activation of the mTOR pathway and the progression of polycystic kidney disease or diabetic nephropathy was reported [29,30,31]. Rapamycin showed protective effects against polycystic kidney disease in animal experiments; however, it is unclear whether rapamycin induces autophagy in polycystic kidney disease [32]. Further precise investigation into the effects of mTOR inhibitors on polycystic kidney disease is needed to enable their clinical application [32]. 

Nakagawa *et al.* [33] demonstrated that the mTOR pathway is activated in the proximal tubular cells of rat kidneys after subtotal nephrectomy and found that treatment with everolimus in rats eight weeks after subtotal nephrectomy, an animal model of end-stage renal disease, had restorative effects on the tubular reabsorption of albumin and the expression levels of membrane transporters in the proximal tubules.

Autophagy is induced in response to renal ischemia-reperfusion and cisplatin induced acute kidney disease [34]. Autophagy-related (ATG)-5 and -7 proximal tubule-specific knockout mice showed more severe renal injury than wild-type mice after ischemia-reperfusion and cisplatin treatment, indicating a protective effect of autophagy during these treatments [34]. mTORC1 inhibitors seem to exert therapeutic effects by inducing autophagy; however, because they also impaired the cell proliferation necessary to recover from damage, caution is advised [34]. In fact, everolimus administration before ischemia-reperfusion increased the signals for kidney injury molecule-1 (a marker for tubular injury) and single-stranded DNA (a marker for apoptotic cells) and decreased that for Ki-67 (a proliferation marker) in the rat proximal tubules, indicating that everolimus diminished renal function after acute tubular injury [35].

**Figure 4:**
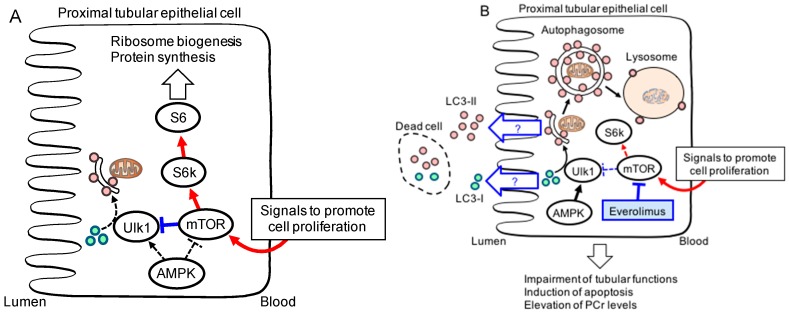
Hypothesized mechanisms of mammalian target of rapamycin (mTOR) inhibitor action in proximal tubular epithelial cells. Rprinted from [35]. Copyright 2012 with permission from Elsevier. (**A**) during the recovery phase of nephrotoxicant-induced tubular injury, the mTOR pathway in proximal tubular cells is activated to promote cell proliferation; (**B**) everolimus treatment exerts antiproliferative effects by blocking cell survival pathways. The quantification of urinary LC3 protein could be beneficial in predicting outcomes of treatment with mTOR inhibitors [36].

On the other hand, urinary LC3 levels were found to be markedly increased after administration of everolimus to rats with ischemia-reperfusion renal injury and cisplatin-induced nephropathy [35]. Because the cytosolic form of LC3-I is modified into its phosphatidylethanolamine-conjugated form, LC3-II, which is associated with autophagosomes, LC3-II has been examined as a marker for autophagic activity [36]. Therefore, the urinary LC3 induced by the administration of everolimus could be a biological marker for mTOR inhibition during acute kidney injury (Figure 4).

## 4. Application of mTOR Inhibitors in Kidney Transplantation

In kidney transplantation, dosage adjustment of mTOR inhibitors sirolimus and everolimus using therapeutic drug monitoring (TDM) is recommended to prevent toxicity of adverse reactions of mTOR inhibitors such as thrombocytopenia, leucopenia, hypercholesterolemia, stomatitis, diarrhea, interstitial pneumonitis, and rejection [12]. The recommended therapeutic range of average sirolimus trough concentrations (*C*_trough_) is set between 5.0 and 15 ng/mL or 6.0 and 12 ng/mL for the conventional immunosuppressive regimens including calcineurin inhibitors tacrolimus and cyclosporine, respectively, but between 10 and 20 ng/mL without calcineurin inhibitors [12]. Toxic *C*_trough_ of sirolimus were established at >15 μg/L [12]. The recommended therapeutic trough range for everolimus in heart transplantation and kidney transplantation in combination with calcineurin inhibitors is between 3 and 8 ng/mL. Correlation between *C*_trough_ and the area under the concentration-time curve (AUC) was examined in kidney transplant patients in a calcineurin inhibitor-free regimen, and *C*_trough_ between 6.0 and 8.0 ng/mL corresponded with an AUC of approximately 120 ng·h/mL. The target concentrations of everolimus in a regimen without calcineurin inhibitor range between 6.0 and 10 ng/mL [12]. A prospective trial with 46 *de novo* kidney transplant patients revealed that the exposure of administered everolimus was not affected regardless of the blood concentration of tacrolimus (1.5–3 ng/mL *vs.* 4–7 ng/mL) [37]. Furthermore, cyclosporine co-administration increased everolimus blood concentration compared to that with tacrolimus co-administration, suggesting the competitive inhibition of everolimus metabolism by cytochrome P450 (CYP) 3A4 [37].

Increased cyclosporine and tacrolimus *C*_trough_ tends to cause nephrotoxicity [38]. Combination therapy with low-dose calcineurin inhibitors and other immunosuppressive drugs is useful to reduce the target concentration of calcineurin inhibitors and avoid calcineurin inhibitor-induced nephrotoxicity [39]. One report showed that an mTOR inhibitor-based protocol was not associated with poorer graft function or higher rejection rates than standard calcineurin inhibitor therapy, but about half of patients (46.8%) receiving mTOR inhibitors were converted to calcineurin inhibitor-based therapy during that study term [40]. The common adverse reactions of mTOR inhibitors are stomatitis and wound-healing problems; therefore, de novo administration of mTOR inhibitors in kidney transplant patients from the early stage is thought to be a risk factor for wound-healing problems [24,41,42]. Future studies evaluating the effectiveness and wound-healing problems of mTOR inhibitors beginning immediately after surgery should be carried out in the future to clarify the potential usefulness of mTOR inhibitors in de novo kidney transplant patients.

mTOR inhibitors in combination with low-dose calcineurin inhibitors also can be safely used in children [39]. Steroidal drugs are well known to interfere with growth in pediatric kidney transplantation, and steroid withdrawal is favorable as early as possible [43]. The pharmacological and toxicological influence of mTOR inhibitors on growth are still controversial in pediatric cases: some studies reported that mTOR inhibitors interfere with growth, whereas others reported no effect [39]. 

## 5. mTOR Inhibitors as Anticancer Drugs

Renal cell carcinoma derived from the proximal tubular cells is one of the malignancies with the poorest prognosis and is highly resistant to chemotherapy [44]. Following cytokine-based immunotherapy, targeted drugs including vascular endothelial growth factor (VEGF) receptor-associated tyrosine kinase inhibitors, the anti-VEGF monoclonal antibody, and mTOR inhibitors were developed as anticancer drugs for renal cell cancer [44]. Because renal clear cell carcinoma, which consists mostly (75%–85%) of kidney tumors, is a highly vascularized malignancy, antiangiogenesis-based therapy is a reasonable approach against this type of cancer [44]. Renal cell carcinoma is associated with loss of function of the von Hippel–Lindau gene [44,45], accumulation of hypoxia-inducible factor (HIF)1α, and increased tumorigenesis [45]. Accumulated HIF1α binds constitutively expressed HIF1β and is translocated into the nucleus, where the HIF complex binds to the HIF-response element of DNA and activates transcription of angiogenic genes, including VEGF-A, epidermal growth factor receptor type 1, platelet-derived growth factor B chain, transforming growth factor α, and erythropoietin [45,46]. mTOR inhibitors prevent cancer progression by inhibiting translation of HIF1α [46]. In treatment of patients with surgical intervention-maladaptive progressing renal clear cell cancer, sunitinib (VEGF receptor-tyrosine kinase inhibitor (VEGFR-TKI)), bevacizumab (anti-VEGF antibody) plus interferon-α, pazopanib (VEGFR-TKI), temsirolimus, everolimus, axitinib (VEGFR-TKI), and sorafenib (VEGFR-TKI) are approved [47,48]. About 40% of patients with renal cell carcinoma develop metastatic renal cell carcinoma (mRCC) [49]. Temsirolimus and VEGFR-TKIs are used as primary treatments against mRCC, and everolimus or axitinib is secondary [49].

To compare the efficacies of everolimus and temsirolimus in patients in mRCC, some studies were carried out. A meta analysis with 863 (92%) patients treated with sunitinib and 74 (8%) with pazopanib or sorafenib as first-line therapy showed that everolimus decreased the risk of death by 26% (hazard ratio (HR), 0.74; 95% confidence interval (CI), 0.59–0.93; *p* = 0.008) and the risk of treatment failure by 30% (HR, 0.70; 95% CI, 0.56–0.88; *p* = 0.002) over temsirolimus [50]. In a retrospective analysis, 90 patients with mRCC (clear cell renal cell carcinoma 87%, non-clear renal cell carcinoma 12%) after progression during first-line VEGFR-TKI therapy were assigned to the second-line temsirolimus treatment group or everolimus treatment group [49]. The median progression-free survival was not different, but overall survival was superior with everolimus compared with temsirolimus (24.2 months *vs.* 12.1 months; HR, 0.58; *p* = 0.047) [49]. Prospective trials are needed to confirm these results; however, everolimus treatment appeared more favorable than temsirolimus in mRCC. On the other hand, some studies raised questions about the efficacy of everolimus as a second-line therapy for renal cancer. For example, a randomized, open-label study compared nivolumab, a programmed death 1 checkpoint inhibitor, with everolimus in patients with advanced clear cell renal cell carcinoma who had received previous treatment [51]. The median overall survival was longer with nivolumab (25 months; 95% CI, 21.8 to not estimable) than with everolimus (19.6 months; 95% CI, 17.6–23.1) [51]. Furthermore, a randomized, open-label study compared cabozantinib, an oral small-molecule inhibitor of tyrosine kinases, with everolimus in patients with advanced clear cell renal cell carcinoma that had progressed after VEGFR-targeted therapy [51]. The estimated median overall survival was longer with cabozantinib (7.4 months; 95% CI, 5.6–9.1) than with everolimus (3.8 months; 95% CI, 3.7–5.4) [52].

## 6. Effect of mTOR Inhibitors on Non-Clear Cell Renal Cell Carcinoma

One-quarter of renal cell carcinoma is non-clear cell renal cell carcinoma; however, the treatment protocol for metastatic non-clear cell renal cell carcinomas is undefined [53]. Sunitinib and everolimus (mTOR inhibitor) are standard first-line and second-line therapies, respectively, in clear cell renal cell carcinoma [54]. Some clinical trials to determine the effect of sunitinib and everolimus on non-clear renal cell carcinoma were carried out [55]. The effectiveness of everolimus for treating non-clear cell renal cell carcinoma was reported, especially in patients with chromophobe renal cell carcinoma, and prior treatment with a VEGF-TKI had no effect on everolimus treatment [56]. A crossover treatment design study showed that everolimus treatment was not inferior to sunitinib as a first-line therapy. The median progression-free survival was 7.9 months for starting with everolimus and 10.7 months for starting with sunitinib (HR, 1.4; 95% CI, 1.2–1.8), and the median combined progression-free survival was 21.1 months for patients switched from everolimus to sunitinib and 25.8 months for patients switched from sunitinib to everolimus (HR, 1.3; 95% CI, 0.9–1.7) [57]. Seventy-three patients with non-clear cell renal cell carcinoma and sarcomatoid clear cell renal cell carcinoma were randomly assigned to receive either everolimus or sunitinib [55]. Progression-free survival in first-line treatment was not significantly different for sunitinib treatment (4.1 months) and everolimus treatment (6.1 months, *p* = 0.6) [55]. However, the median overall survival was significantly higher with first-line sunitinib treatment (not reached *vs.* 10.5 months; *p* = 0.014); therefore, following the recommendation of the data and safety monitoring committee, the study was prematurely terminated [54]. An open-label randomized study of 108 patients with metastatic papillary, chromophobe, or unclassified non-clear cell renal cell carcinoma revealed that sunitinib improved progression-free survival (8.3 months; 80% CI, 5.8–11.4) compared with everolimus (5.6 months [5.5–6.0]) (HR, 1.41; 80% CI, 1.03–1.92; *p* = 0.16) [53]. Consequently, everolimus was not recommended as a first-line therapy in non-clear cell renal cell carcinoma; however, the effect of sunitinib effect was very modest, and development of a more effective therapy for non-clear cell renal cell carcinomas is needed [54,55].

## 7. Effect of mTOR Inhibitors on Diabetic Nephropathy

Blood glucose level monitoring is recommended in patients receiving treatment with mTOR inhibitors because mTOR inhibitors are accompanied by a high incidence of hyperglycemia and new-onset diabetes (13%–50%) when used as anticancer drugs [58]. Although the clinical data are not sufficient, animal data suggest that the mechanisms responsible for hyperglycemia with mTOR inhibitors are likely due to the combination of impaired insulin secretion and insulin resistance [58]. Hyperglycemia is essential for the onset of diabetic nephropathy, which is a major factor of end-stage renal disease [59]. Hyperglycemia induces an unusual activation of protein kinase C and is involved in the development of diabetic nephropathy. It was associated with TGF-β1, fibronectin, and α1 (IV) collagen production in renal glomeruli of diabetic rats [60]. Furthermore, hyperglycemia is the cause of advanced glycosylation end product (AGE) generation in patients with diabetes. AGEs stimulate intrinsic glomerular cells to produce TGF-β1, which contributes to glomerular sclerosis and tubulointerstitial damage by inducing abnormal extracellular matrix production [59]. Though mTOR inhibitors are one risk factor for diabetic nephropathy because they cause hyperglycemia, researchers reported their efficacy in treating diabetic nephropathy [61,62,63,64,65]. Sirolimus inhibited glucose-induced phosphorylation of p70S6 kinase and its substrate, S6 ribosomal protein, in mesangial cells, and inhibition of mTOR with sirolimus attenuated the morphological and functional disorders of diabetic kidneys in a type 2 diabetes mouse model [65].

Apoptosis contributes to the development of diabetic nephropathy, and hyperglycemia triggers the generation of free radicals and oxidant stress in tubular cells. An *in vitro* experiment using a proximal tubular epithelial cell line demonstrated that high glucose-induced peroxynitrate leads caspase-mediated apoptosis [64]. The tuberin/mTOR pathway was implicated in apoptosis of tubular epithelial cells in diabetes. Tuberin is a suppressor of mTOR, and phosphorylated tuberin loses its suppressive effect against mTOR [63]. High glucose treatment increased phosphorylation of tuberin and p70S6K, phosphorylation of Bcl-2, expression of cytosolic cytochrome c, and caspase 3 activity in proximal tubular epithelial cells. Activated caspase 3 likely promoted Ying Yang1 nuclear translocation and enhanced cleavage of poly (ADP-ribose) polymerase, resulting in apoptosis [62]. Those effects were inhibited by rapamycin pretreatment, and the number of apoptotic cells induced by high glucose was decreased [62].

The mTOR signaling pathway is important in podocyte homeostasis [66]. Analysis of mRNA extracted from the microdissected glomeruli of patients with glomerulopathies showed that expression of mTOR mRNA and mTORC1 target genes increased in very early diabetic nephropathy [66]. In glomerular disease, short-term activation of mTOR signaling has a positive effect, but long-term activation leads to proteinuria and glomerulosclerosis [66]. Lack of mTORC1 and mTORC2 in mouse podocytes aggravated glomerular lesions, but downregulating mTORC1 expression in podocytes prevented glomerulosclerosis and ameliorated the progression of glomerular disease in diabetic nephropathy [66]. Rapamycin treatment prevented glomerular hypertrophy, glomerular basement membrane thickening, mesangial expansion, podocyte loss, macrophage infiltration, and albuminuria in type-2 diabetic model db/db mice [67]. Furthermore, non diabetic young and adult mice with podocyte specific mTORC1 activation showed many diabetic nephropathy features, including podocyte loss, glomerular basement membrane thickening, mesangial expansion, and proteinuria [67]. Genetic reduction of podocyte-specific mTORC1 in diabetic animals suppressed the development of diabetic nephropathy [67]. These studies suggested that partial suppression of the mTORC1 pathway might represent a therapeutic strategy to prevent diabetic nephropathy without compromising podocyte homeostasis.

Although the precise mechanism remains unclear, involvement of innate immunity in the early stage of diabetic nephropathy associated with mTOR was reported. Involvement of the toll-like receptor (TLR)4-mediated pathway in promoting tubulointerstitial inflammation in diabetic nephropathy was suggested. In the renal tubules of patients with diabetic nephropathy, TLR4 is upregulated, but TLR2 is not, and TLR4 deletion attenuated albuminuria, renal dysfunction, and renal cortical NF-κB in streptozotocin-induced diabetic mice [61]. Sirolimus inhibited upregulated TLR4 and pro-inflammatory factor interleukin 17 in early-stage diabetic nephropathy in streptozotocin-induced diabetic rats [61].

## 8. Conclusions

mTOR inhibitors are useful for treating various renal diseases. Further study is needed to exploit more favorable therapeutic strategies for renal disease with mTOR inhibitors including rapalogues and ATP-competitive inhibitors of mTOR.
